# Supplementary material for: Multi-omics single-cell analysis reveals key regulators of HIV-1 persistence and aberrant host immune responses in early infection
Source: eLife. 2025 Aug 21;14:RP104856. doi: 10.7554/eLife.104856 (PMC12370253; doi:10.7554/eLife.104856)
Supplement: Supplementary file 1. — (A) Epidemiological and virological characteristics, and sequencing data information for nine acute HIV-infected patients at baseline. (B) Differentially expressed genes overlapped with KLF2 target gene in CD4 T cell (HIV-1 RNA+ vs. uninfected). (C) Primers used for HIV-1 targeted sequencing. [file elife-104856-supp1.docx]

**Supplementary Tables**

| Sample ID | Sex | Age | Duration from HIV infection (Day) | CD4+ T  (cells/mm3) | HIV-1  Viral loads  (Log10 copies/ml) | HIV-1 p24 Antigen | HIV-1  Antibodies  (PA) | Sequencing  Techniques | | |
| --- | --- | --- | --- | --- | --- | --- | --- | --- | --- | --- |
|  |  |  |  |  |  |  |  | scRNA-seq | scATAC-seq | sc-Multiome |
| 06-D10629 | M | 51 | 158 | 760 | 16.1 | Positive | Negative | No | No | Yes |
| C1-C80404 | M | 17 | 0 | 443 | 13.4 | Positive | Negative | Yes | No | Yes |
| 35-C90552 | M | 45 | 20 | 655 | 13.2 | Positive | Negative | Yes | No | Yes |
| 36-C70518 | M | 28 | 3 | N/A | 14.9 | N/A | N/A | Yes | No | Yes |
| 02-C80622 | M | 28 | 0 | 970 | 16.1 | Positive | Negative | Yes | Yes | No |
| 02-D00365 | M | 34 | 12 | 482 | 15 | Negative | Negative | Yes | Yes | No |
| 36-C80800 | F | 50 | 0 | 643 | 16.1 | Positive | Negative | Yes | Yes | No |
| 36-D00564 | M | 50 | 175 | 888 | 13.3 | Negative | Positive | Yes | Yes | No |
| 02-C80649 | M | 59 | 0 | 597 | 15.7 | N/A | N/A | Yes | No | No |

* M: Male, F: Female

**Table A. Epidemiological and Virological Characteristics, and Sequencing Data Information for 9 Acute HIV-infected Patients at Baseline**

| **Gene** | **p_val** | **avg_logFC** | **pct.1** | **pct.2** | **p_val_adj** |
| --- | --- | --- | --- | --- | --- |
| TPT1 | 2.17E-74 | 0.664730297 | 0.887 | 0.829 | 6.97E-70 |
| VIM | 4.75E-44 | 0.486947637 | 0.711 | 0.507 | 1.52E-39 |
| TMSB10 | 1.01E-67 | 0.484596487 | 0.924 | 0.821 | 3.25E-63 |
| CD37 | 7.61E-57 | 0.483621046 | 0.615 | 0.344 | 2.44E-52 |
| HMGB1 | 1.26E-55 | 0.449454734 | 0.786 | 0.554 | 4.05E-51 |
| LTB | 4.33E-46 | 0.44052761 | 0.839 | 0.644 | 1.39E-41 |
| TXNIP | 2.55E-64 | 0.408496641 | 0.955 | 0.843 | 8.16E-60 |
| IL32 | 2.84E-29 | 0.400628464 | 0.723 | 0.559 | 9.10E-25 |
| KLF2 | 2.62E-45 | 0.383897324 | 0.777 | 0.546 | 8.41E-41 |
| PNRC1 | 1.45E-30 | 0.299190983 | 0.651 | 0.434 | 4.64E-26 |
| ACAP1 | 5.87E-25 | 0.270001786 | 0.553 | 0.358 | 1.88E-20 |
| IL2RG | 2.18E-25 | 0.259545545 | 0.413 | 0.233 | 7.00E-21 |
| SF1 | 3.40E-16 | 0.211089385 | 0.8 | 0.639 | 1.09E-11 |
| TBC1D10C | 1.01E-15 | 0.208888529 | 0.382 | 0.244 | 3.23E-11 |
| PIM1 | 2.00E-14 | 0.207804659 | 0.361 | 0.233 | 6.42E-10 |
| SIGIRR | 3.57E-13 | 0.198079587 | 0.321 | 0.203 | 1.14E-08 |
| CCND2 | 5.82E-12 | 0.189860451 | 0.311 | 0.198 | 1.87E-07 |
| HNRNPH1 | 1.00E-15 | 0.188659263 | 0.776 | 0.62 | 3.22E-11 |
| TSC22D3 | 9.49E-15 | 0.171356073 | 0.467 | 0.317 | 3.04E-10 |
| DNAJC19 | 3.04E-17 | 0.167563816 | 0.198 | 0.095 | 9.74E-13 |
| CITED4 | 3.31E-16 | 0.1675141 | 0.206 | 0.103 | 1.06E-11 |
| UBE2V1 | 1.21E-14 | 0.14695742 | 0.121 | 0.05 | 3.89E-10 |
| JUND | 1.38E-15 | 0.140976983 | 0.559 | 0.383 | 4.43E-11 |
| MKRN1 | 1.04E-05 | 0.13071128 | 0.245 | 0.179 | 0.333091 |
| PRDM1 | 2.48E-05 | 0.122195235 | 0.117 | 0.074 | 0.796627 |
| BNIP3 | 3.35E-11 | 0.116108557 | 0.119 | 0.056 | 1.07E-06 |
| LINC-PINT | 0.008899725 | 0.071251147 | 0.394 | 0.341 | 1 |
| TCEA2 | 0.000497195 | 0.068589737 | 0.048 | 0.026 | 1 |
| PYCR2 | 2.40E-07 | 0.060669396 | 0.119 | 0.067 | 0.00771 |
| KLF9 | 0.001952912 | 0.058854945 | 0.21 | 0.163 | 1 |
| CNIH1 | 1.34E-07 | 0.056829405 | 0.125 | 0.07 | 0.004296 |
| ARF6 | 0.000182984 | 0.054848756 | 0.208 | 0.154 | 1 |
| BHLHE40 | 0.000411762 | 0.053459996 | 0.058 | 0.033 | 1 |
| RPS6KA1 | 0.024616225 | 0.044512378 | 0.088 | 0.066 | 1 |
| CSNK2A1 | 0.002730067 | 0.038988524 | 0.189 | 0.146 | 1 |
| PAWR | 0.000829052 | 0.036027924 | 0.018 | 0.007 | 1 |
| FOXJ2 | 0.830108419 | 0.025027762 | 0.023 | 0.025 | 1 |
| SOCS3 | 0.012105249 | 0.017691013 | 0.143 | 0.111 | 1 |
| NRP2 | 0.188390647 | 0.010845121 | 0.003 | 0.001 | 1 |
| DPYSL4 | 0.001187095 | 0.010504532 | 0.003 | 0 | 1 |
| KDM5B | 0.355956849 | 0.006549433 | 0.132 | 0.12 | 1 |
| NANOG | 0.181105411 | 0.005793679 | 0.002 | 0.001 | 1 |
| PHC1 | 0.69964956 | 0.005013823 | 0.035 | 0.032 | 1 |
| MYCL | 0.344726739 | 0.000883179 | 0.001 | 0 | 1 |
| S1PR3 | 0.744460326 | -0.000627781 | 0.001 | 0.001 | 1 |
| DNMT3A | 0.489967984 | -0.001010646 | 0.141 | 0.15 | 1 |
| RRP1B | 0.205136712 | -0.001104724 | 0.163 | 0.143 | 1 |
| NXNL2 | 0.450035052 | -0.001541665 | 0 | 0.001 | 1 |
| DMRT1 | 0.450035052 | -0.001673579 | 0 | 0.001 | 1 |
| BMP7 | 0.450035052 | -0.001896741 | 0 | 0.001 | 1 |
| SNAI1 | 0.450035052 | -0.001985345 | 0 | 0.001 | 1 |
| MFSD2A | 0.354768244 | -0.002548496 | 0 | 0.001 | 1 |
| ZMYM3 | 0.958216028 | -0.003288628 | 0.02 | 0.021 | 1 |
| PLA2G1B | 0.450035052 | -0.003297112 | 0 | 0.001 | 1 |
| NOTCH3 | 0.450035052 | -0.004169338 | 0 | 0.001 | 1 |
| TRPM3 | 0.613033626 | -0.006833198 | 0.001 | 0.002 | 1 |
| RAD51C | 0.052466005 | -0.010661489 | 0.053 | 0.038 | 1 |
| CDKN1A | 0.090790919 | -0.013386654 | 0 | 0.003 | 1 |
| DENND2A | 0.054112543 | -0.014882968 | 0.001 | 0.006 | 1 |
| HOXB4 | 0.634292218 | -0.017283224 | 0.015 | 0.017 | 1 |
| RABIF | 0.486560265 | -0.025469725 | 0.029 | 0.034 | 1 |
| RPN1 | 0.438232729 | -0.026026513 | 0.119 | 0.108 | 1 |
| KLF4 | 0.157228999 | -0.028322843 | 0 | 0.002 | 1 |
| AP1M1 | 0.66257167 | -0.032616089 | 0.093 | 0.098 | 1 |
| HOOK2 | 0.214501657 | -0.033820571 | 0.056 | 0.066 | 1 |
| PGS1 | 0.765659484 | -0.056894937 | 0.061 | 0.057 | 1 |
| TGIF1 | 0.68822548 | -0.06021225 | 0.035 | 0.037 | 1 |
| AP5M1 | 0.282209538 | -0.061633796 | 0.09 | 0.101 | 1 |
| SP1 | 0.372255551 | -0.069005677 | 0.057 | 0.064 | 1 |
| ATG4C | 0.014615343 | -0.080024748 | 0.021 | 0.037 | 1 |
| PXN | 0.083160525 | -0.09286964 | 0.118 | 0.137 | 1 |
| DLGAP1 | 0.000585314 | -0.110979077 | 0.003 | 0.018 | 1 |
| TEX14 | 3.59E-08 | -0.127037624 | 0.005 | 0.041 | 0.001152 |
| CD83 | 0.000206579 | -0.129834592 | 0.006 | 0.025 | 1 |
| CYLD | 0.003516763 | -0.141271159 | 0.531 | 0.546 | 1 |
| SMARCAD1 | 0.000407205 | -0.201335112 | 0.066 | 0.102 | 1 |
| MSRA | 0.000171836 | -0.232231502 | 0.059 | 0.095 | 1 |
| FOS | 0.000404187 | -0.233427767 | 0.032 | 0.061 | 1 |
| RAD23B | 6.14E-05 | -0.238391059 | 0.135 | 0.182 | 1 |
| ARL8B | 2.25E-06 | -0.24180768 | 0.08 | 0.133 | 0.072268 |
| JUNB | 0.000379409 | -0.274886806 | 0.504 | 0.519 | 1 |
| JARID2 | 6.74E-08 | -0.291366599 | 0.148 | 0.221 | 0.002162 |
| RYBP | 3.05E-10 | -0.329940877 | 0.062 | 0.133 | 9.78E-06 |
| ATG5 | 4.10E-10 | -0.377720823 | 0.105 | 0.18 | 1.32E-05 |
| SLC2A3 | 2.14E-11 | -0.406462164 | 0.143 | 0.235 | 6.87E-07 |
| PPP1R12A | 2.37E-14 | -0.415261823 | 0.319 | 0.426 | 7.60E-10 |
| DTNB | 4.43E-21 | -0.496467883 | 0.044 | 0.158 | 1.42E-16 |
| KANK1 | 5.65E-20 | -0.543001768 | 0.042 | 0.15 | 1.81E-15 |

**Table B. Differentially expressed genes overlapped with KLF2 target gene in CD4 T cell. (HIV-1 RNA+ vs. Uninfected)**

| Primer name | sequence (5'-3') |
| --- | --- |
| P1 | GAAATCTGTTGACTCAGATTGGTTGCACTTTAAATTTTCCCATTAGCC |
| P2 | CTATGGCAGGAAGAAGCGGAGACAGCGACGAAGAGCTCCTCA |
| P3 | CTACAAGGGACTTTCCGCTGGGGACTTTCCAGGGAGGCGTGG |

**Table C. Primers used for HIV-1 targeted sequencing**
